# Labor patterns of spontaneous first-stage labor in Chinese women with normal neonatal outcomes

**DOI:** 10.1371/journal.pone.0305243

**Published:** 2024-07-03

**Authors:** Li Peng, Zengyu Chen, Shiyu Weng, Jian Huang, Mei Peng, Yali Deng, Ying Xu, Fangfang Zhou, Yamin Li

**Affiliations:** 1 Clinical Nursing Teaching and Research Section, The Second Xiangya Hospital of Central South University, Changsha, Hunan, China; 2 Department of Obstetrics and Gynecology, The Second Xiangya Hospital of Central South University, Changsha, Hunan, China; 3 Xiangya School of Nursing, Central South University, Changsha, Hunan, China; 4 The Second Xiangya Hospital of Central South University, Changsha, Hunan, China; Universidade Federal de Minas Gerais, BRAZIL

## Abstract

**Background:**

Friedman’s standards, developed almost 50 years ago, may no longer align with the needs of today’s obstetric population and current pregnancy management practices. This study aims to analyze contemporary labor patterns and estimate labor duration in China, focusing on first-stage labor data from Chinese parturients with a spontaneous onset of labor.

**Methods:**

This retrospective observational study utilized data from electronic medical records of a tertiary hospital in Changsha, Hunan. Out of a total of 2,689 parturients, exclusions were made for multiple gestations, preterm, post-term, or stillbirth, cesarean delivery, non-vertex presentation, and neonatal intensive care unit admission. Average labor curves were constructed by parity using repeated-measure analysis, and labor duration was estimated through interval-censored regression, stratified by cervical dilation at admission. We performed an analysis to assess the impact of oxytocin augmentation and amniotomy on labor progression and conducted a sensitivity analysis using women with complicated outcomes.

**Results:**

Nulliparous women take over 180 minutes for cervical dilation from 3 to 4 cm, and the duration from 5 to 6 cm exceeds 145 minutes. Multiparous women experience shorter labor durations than nulliparous. Labor acceleration is observed after 5 cm in nulliparous, but no distinct inflection point is evident in the average labor curve. In the second stage of labor, the 95th percentile for nulliparous, with and without epidural analgesia, is 142 minutes and 127 minutes, respectively.

**Conclusions:**

These findings provide valuable insights for the reassessment of labor and delivery processes in contemporary obstetric populations, including current Chinese obstetric practice.

## Introduction

Abnormal labor progression is an event to be alerted to in obstetric practice. The definition of abnormal labor progression affects how it is observed and managed. In the 1970s, Friedman provided a definition for abnormal labor progression during the active phase, characterizing it as cervical dilation of less than 1.2 cm per hour in nulliparous women and less than 1.5 cm per hour in multiparous women. The absence of significant changes in cervical dilation when the uterus is adequately contracted for >2 hours was considered labor arrest according to Friedman’s criteria [1]. Furthermore, Friedman also outlined the labor process into several stages and phases and depicted a labor curve [2, 3]. These definitions from the 1970s continue to influence contemporary approaches to labor management.

With the improvement in the quality of life, contemporary demographic and obstetric factors for pregnant women have undergone changes, such as an increase in maternal age and weight, gestational weight, and neonatal birth weight [4–6]. Additionally, due to technological advancements, obstetric interventions (i.e., epidural analgesia, amniotomy, and the use of oxytocin) have become more prevalent, potentially altering the natural progression of labor [7, 8]. Recent research has proposed that the progression of the first stage of labor and the threshold separating latent and active phases may differ from what previous studies suggested [9–12]. These observations are also reflected in the current World Health Organization (WHO) recommendations [13]. Therefore, Friedman’s standard may no longer fully align with the need of the contemporary obstetric population and the current management of pregnant women [14].

In 2010, Zhang et al. [15] conducted a study using data from a large number of contemporary parturients across the U.S. to examine the patterns of spontaneous labor with normal neonatal outcomes. They found that after reaching 6 cm of cervical dilation, the rate of dilation accelerated, and the progression from 4 to 6 cm was much shorter than previously thought. However, it’s noteworthy that only 3% of the study population was Asian or Pacific Islanders, leading to a lack of representation of Asians in their findings. Considering the unique characteristics of Asian obstetric practice, labor patterns derived from Western data may not be universally applicable. Although Lu et al. [16] analyzed labor patterns in 3,079 Asian American women using the same dataset, there is still a lack of contemporary research specifically focused on labor patterns in East Asian women. To our knowledge, only two studies have examined the contemporary patterns of spontaneous labor in East Asian parturients (one examined labor patterns in Japanese nulliparous and multiparous, and one examined labor patterns in Chinese nulliparous) [17, 18]. Further evidence is needed to fully understand contemporary patterns of spontaneous labor among East Asian women.

To fill this gap, this study examined labor patterns and estimated labor duration in China using contemporary labor data of nulliparous and multiparous with spontaneous onset of the first stage of labor.

## Materials and methods

### Sample and procedure

In this retrospective observational study, we used detailed labor and delivery data from electronic medical records in a Chinese university-affiliated tertiary hospital located in Changsha, Hunan from January 1, 2017, to December 31, 2019. Approval to access electronic medical records for research purposes was granted on January 18, 2023. Upon admission, patients were informed that their information would be recorded in electronic medical records with guaranteed privacy protection. Written consent was sought regarding the use of their data in scientific research. In the absence of consent, their data would be classified separately in electronic medical records, and no research could be conducted using this part of the data. It was clarified during the informed consent process that their decision would not impact their hospital treatment. Throughout data cleaning and analysis, the authors maintained the anonymity of individual participants by avoiding the use of personal identifiers. We conducted a comprehensive data extraction process that included detailed information on maternal demographic characteristics, medical history, reproductive history, prenatal history, and labor and delivery summary. To ensure accurate and complete newborn status, we linked the newborn records with information from the neonatal intensive care unit (NICU). Furthermore, we leveraged data from the electronic labor database to obtain repeated and time-stamped cervical dilations, which provided insights into labor progression.

**Fig 1:**
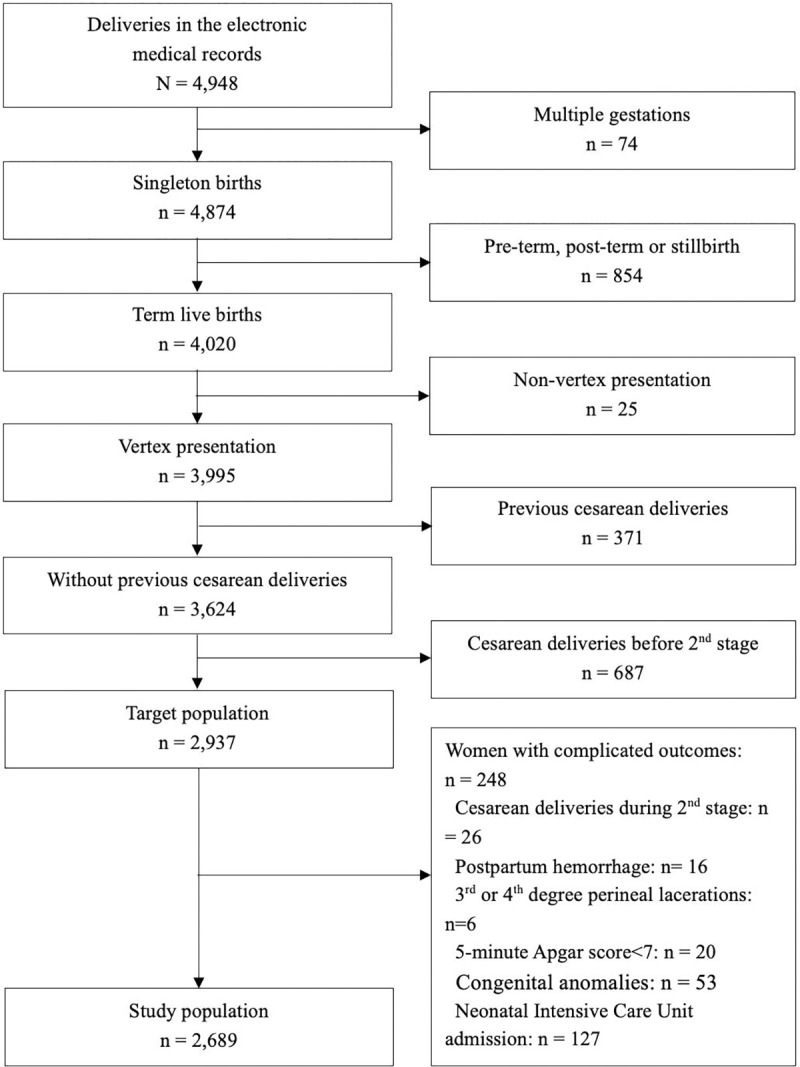
Flow diagram of the study selection process.

Exclusion criteria comprised parturients with multiple gestations, pre-term or post-term deliveries, stillbirth, and non-vertex presentation. Additionally, cesarean deliveries before the second stage of labor, previous cesarean deliveries, multiple births from the same multiparous, and women with induced labors were excluded from the data analysis. To establish labor patterns in parturients without complicated outcomes, we further excluded women with complicated outcomes, which involving cesarean delivery during the second stage, postpartum hemorrhage, 3^rd^ or 4^th^ degree perineal lacerations, and adverse neonatal outcomes (newborns with 5-minute Apgar scores <7, congenital anomalies or NICU admission), from the main analysis. Ultimately, a total of 4,948 parturient were extracted from the database. Among these, 2,689 women who had a singleton term pregnancy with spontaneous onset of labor, vertex presentation, vaginal delivery, and without complicated outcomes were included in this study, referred to as the "study population" (Fig 1). This project was approved by the ethics committee of the National Clinical Research Center in the Second Xiangya Hospital, China (2022JJ30802). All methods were carried out in accordance with relevant guidelines and regulations in the Declaration of Helsinki.

### Statistical analysis

In this study, pregnant women were categorized based on parity (nulliparous or multiparous). Statistical differences in baseline characteristics between these two groups were analyzed. We calculated mean and standard deviation and used t-tests for continuous data with a normal distribution. Median, 10^th^ and 90^th^ percentiles were calculated, and Wilcoxon rank-sum tests were conducted for continuous variables with a skewed distribution. For categorical variables, percentage and chi-square tests were used.

In order to construct average labor curves by parity, a repeated-measure analysis was conducted using the 7th-degree polynomial model [19]. In this analysis, we set the starting point at the first time when cervical dilation reached 10 cm (time = 0), and the time was calculated backward (e.g., 60 minutes before complete dilation = -60 minutes). We reverted the x-axis (time) to a positive value once the labor curve models had been computed (i.e., a -180 to 0 minutes range has been changed to a 0 to 180 minutes range).

With the assumption that the labor data are log-normally distributed [20], we used an interval-censored regression to estimate the duration of labor based on the time it took for each integer centimeter of dilation to progress to the next (called "traverse time") [20]. The median and 95th centile were calculated. As multiparous women were often admitted later than nulliparous women, many did not have measures of cervical dilation before 4 cm. Consequently, the calculation of time duration in multiparous women commenced at 4 cm, unlike nulliparous women, whose calculation began at 3 cm. To assess the impact of oxytocin augmentation and amniotomy on labor progression, we performed subgroup analyses by comparing the labor interval between women who received oxytocin augmentation and those who did not, as well as between women who underwent amniotomy and those who did not. Additionally, we conducted a sensitivity analysis using the target population (comprising the study population plus women with complicated outcomes) to mitigate selection bias during the extraction process and check the generalizability of labor patterns from the main analysis [21].

Finally, we calculated the cumulative duration of labor from admission to the first 10 cm in nulliparous. This cumulative duration could address the clinical experience where women are observed at a given dilation and then measured periodically. Recognizing that pregnant women admitted at different dilation levels may progress through labor differently, we provided estimates based on their dilation at admission (2 cm, 3 cm, and 4 cm). Then, we depicted a partograph using the 95th percentile of the duration of labor during the first stage, serving as a critical value to draw the ’dystocia line’ in the staircase curve. Statistical analyses and figure depiction were conducted using Stata 16.0 software (StataCorp, College Station, Texas, USA).

## Results

A total of 2,689 Chinese women were included in the main data analysis, with 1,478 and 1,211 parturients in parity groups 0 and 1+, respectively (Table 1). Maternal age increased with higher parity, while the body mass index showed a decreasing trend. The average gestational age at delivery was 39.0 weeks for parity 0 and 38.8 weeks for parity 1+. The use of oxytocin was more common among nulliparous women. Less than 5% of parturients used epidural analgesia for labor pain, and approximately 30% of women experienced amniotomy during the first stage of labor. The characteristics of nulliparous and multiparous women, both with and without oxytocin augmentation and amniotomy, are shown in S1, S2 Tables in S1 File.

**Table 1:** Characteristics of the study population by parity (*N* = 2,689).

	Nulliparous (*n* = 1478)	Multiparous (*n* = 1211)	*P*-value
Maternal age (mean ± SD, years)	28.3 ± 3.3	32.4 ± 4.2	<0.001
Maternal weight (mean ± SD, kg)	68.0 ± 8.0	67.4 ± 7.0	0.023
Maternal height (mean ± SD, cm)	161.0 ± 4.6	160.8 ± 4.4	0.180
BMI at admission (mean ± SD, kg/m^2^)	26.2 ± 2.8	26.0 ± 2.3	0.055
Cervical dilation at admission (cm) [median, 10^th^, 90^th^ centiles]	3 [1, 4]	3 [2, 4]	0.005
Oxytocin use (%)	18.8	11.6	<0.001
Epidural analgesia (%)	3.9	1.1	<0.001
Amniotomy (%)	32.6	26.8	0.001
Total number of vaginal exams in 1^st^ stage [median, 10^th^, 90^th^ centiles]	4 [3, 6]	4 [3, 5]	<0.001
Gestational age at delivery (mean ± SD, weeks)	39.0 ± 1.0	38.8 ± 1.0	<0.001
Birthweight (mean ± SD, grams)	3274 ± 333	3344 ± 346	<0.001

**Fig 2:**
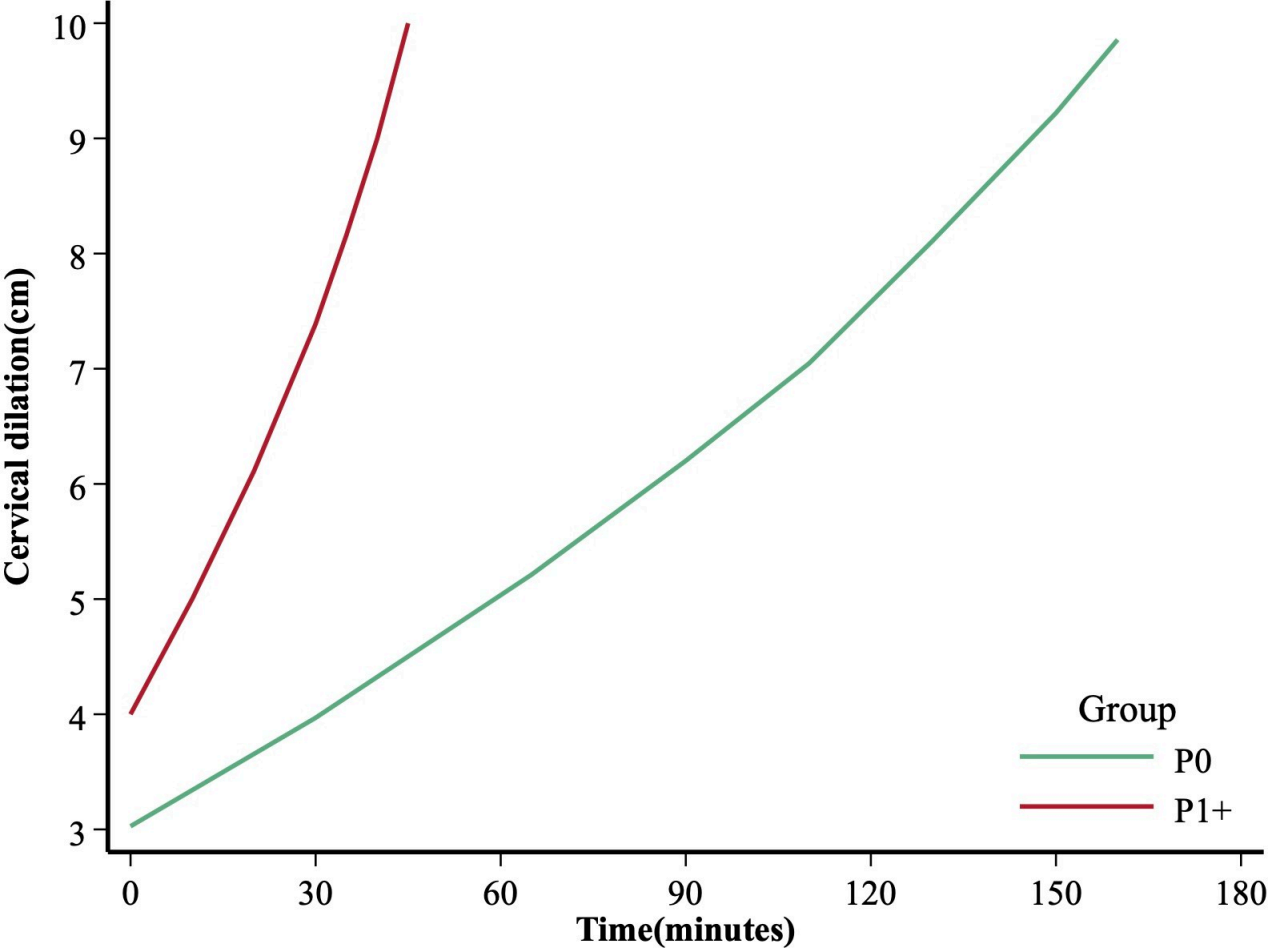
Average labor curves among study population by parity. P0: nulliparous; P1+: multiparous.

The duration of the first stage of labor, from one centimeter of dilation to the next, is shown in Table 2. In nulliparous, the 95th centile indicates that at 3 cm, progression to 4 cm could take up to 180 minutes, while at 4 cm, it may take 145 minutes to progress to 5 cm. The median and 95th centile of the full duration of the first stage of labor (4–10 cm) in nulliparous were longer than those in multiparous, aligning with the average labor curves. Notably, labor acceleration occurred when the cervix reached 5 cm in nulliparous women. The table also suggests that, after cervical dilation is greater than 6 cm, the 95th percentiles of time intervals were less than 60 minutes in nulliparous women and less than 30 minutes in multiparous women. In the second stage of labor, the 95th percentile for nulliparous, with and without epidural analgesia, was 142 minutes and 127 minutes, respectively. Multiparous had much shorter durations in the second stage. Fig 2 depicts the average labor curves for various parties. The time intervals of 1st-stage were about 175 and 45 minutes in nulliparous (3–10 cm) and multiparous (4–10 cm), respectively. The average labor curve for nulliparous did not show a clear inflection point.

**Table 2:** Duration of labor (in hours) for study population by parity.

Cervical dilation (cm)	Nulliparous		Multiparous	
	*n*	Median (95^th^ centile)	*n*	Median (95^th^ centile)
3–4	1145	45 (180)	--	--
4–5	1376	30 (145)	1148	10 (50)
5–6	1436	20 (90)	1186	10 (30)
6–7	1465	15 (60)	1201	5 (20)
7–8	1468	10 (60)	1203	5 (20)
8–9	1469	10 (60)	1204	5 (15)
9–10	1471	15 (45)	1204	5 (15)
3–10	1145	175 (510)	--	--
4–10	1376	117.5 (370)	1148	45 (125)
2^nd^ stage with epidural	58	71.5 (142)	13	21 (79)
2^nd^ stage without epidural	1420	50 (127)	1198	17 (58)

S3 Table in S1 File presents the median and 95th centile of the first-stage labor in women with and without oxytocin use and S4 Table in S1 File shows the median and 95th centile of the first-stage labor in women with and without amniotomy, categorized by parity. For nulliparous women, the median time intervals of labor from 3 to 10 cm were 220 and 168 minutes with and without oxytocin, respectively, while the 95th centiles were 550 and 490 minutes, respectively. Time intervals for the first stage of labor were shorter in nulliparous women without oxytocin compared to those with. This phenomenon is also found in the subgroup analysis according to amniotomy (nulliparous with amniotomy vs. nulliparous without amniotomy: 3-10cm median time, 240 minutes vs. 140 minutes; 3-10cm 95th centile time, 600 minutes vs. 430 minutes). In multiparous women, the time duration (4–10 cm) appeared almost the same regardless of oxytocin use and were faster in those without amniotomy than those with amniotomy. According to the average labor curves for subgroup analysis regarding oxytocin use (S1 Fig in S1 File), the time intervals of the first stage were about 220 and 45 minutes in nulliparous and multiparous with oxytocin, respectively. In women without oxytocin, the time duration was about 170 minutes in nulliparous and 45 minutes in multiparous. The average labor curve for nulliparous women did not exhibit a clear inflection point, and the slope of the curve for nulliparous women without oxytocin was steeper than for those with oxytocin. S2 Fig in S1 File showed the average labor curves for subgroup analysis regarding amniotomy. The slope of the average labor curve for study population without amniotomy was steeper than those with amniotomy, both in nulliparous and multiparous.

Sensitivity analysis was conducted using the target population, which included women without complicated outcomes and those with complicated outcomes. The characteristics of the target population, categorized by parity, are presented in S5 Table in S1 File. In nulliparous women, the 95th centile time intervals for the first stage of labor were longer in women with complicated outcomes than nulliparous in target population. In addition, the duration of first stage among nulliparous in the target population were longer than nulliparous in the study population. In multiparous women, the time duration appeared similar between the overall multiparous group and multiparous women with complicated outcomes until 8 cm or later, when the latter progressed notably faster than the overall multiparous group (Table 3). S2 Fig in S1 File illustrates that there are also no obvious inflection points in the labor curves of nulliparous without normal outcomes.

**Table 3:** Duration of labor (in minutes) for the target population and parturient with complicated outcomes by parity.

Cervical dilation (cm)	Nulliparous in target population		Multiparous in target population		Nulliparous with complicated outcomes		Multiparous with complicated outcomes	
	*n*	Median (95^th^ centile)	*n*	Median (95^th^ centile)	*n*	Median (95^th^ centile)	*n*	Median (95^th^ centile)
3–4	1284	45 (210)	--	--	139	60 (290)	--	--
4–5	1520	30 (150)	1224	10 (50)	144	30 (210)	76	15 (50)
5–6	1583	20 (90)	1266	10 (30)	147	20 (120)	80	10 (30)
6–7	1609	15 (60)	1281	5 (20)	144	20 (105)	80	5 (20)
7–8	1608	10 (60)	1283	5 (20)	140	15 (90)	80	5 (20)
8–9	1609	10 (60)	1284	5 (15)	140	15 (60)	80	5 (12.5)
9–10	1610	15 (45)	1283	5 (15)	139	15 (60)	79	5 (10)
3–10	1259	180 (520)	--	--	114	192.5 (540)	--	--
4–10	1504	120 (375)	1223	45 (125)	128	135 (480)	75	40 (135)
2^nd^ stage with epidural	64	68 (126)	13	21 (79)	6	22 (123)	0	--
2^nd^ stage without epidural	1581	50 (130)	1279	17 (60)	161	52 (149)	81	19 (72)

**Fig 3:**
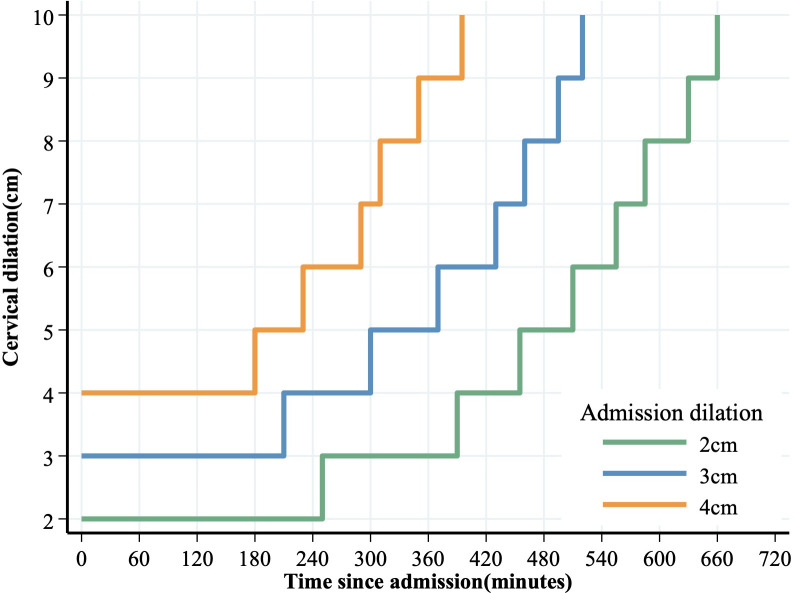
The 95th centile of cumulative duration of labor from admission among singleton, term nulliparous with spontaneous onset of labor, vertex presentation, vaginal delivery, and normal neonatal outcomes.

The partograph defined dystocia as the slowest 5% of nulliparous (Fig 3). Each specific cervical dilation at admission (2, 3, and 4 cm) has its staircase curve extending until reaching 10 cm. In nulliparous women with a spontaneous onset of labor, adequate labor progress is defined when a labor curve stays on the left side of the staircase curve. During the first stage, if a nullipara’s labor crosses the 95th percentile limit (i.e., shifts to the right side of the curve), her labor may be considered prolonged, signaling a potential need for intervention.

## Discussion

Our study utilized data from the electronic medical records from a Chinese tertiary hospital in Changsha, Hunan, focusing on contemporary parturients in China with specific criteria: singleton term pregnancy, spontaneous onset of labor, vertex presentation, vaginal delivery, and normal neonatal outcomes. Our findings showed that nulliparous may experience labor durations exceeding 180 minutes to progress from 3 to 4 cm dilation and over 145 minutes to progress from 4 to 5 cm dilation. Although there was a slight acceleration when the cervix reached 5 cm in nulliparous women, no distinct inflection point was observed in the average labor curves. In the second stage of labor, the 95th percentile for nulliparous with and without epidural analgesia was 142 minutes and 127 minutes, respectively. Additionally, we performed subgroup analyses to assess the impact of oxytocin augmentation and amniotomy on labor progression and conducted a sensitivity analysis using women with complicated outcomes. Finally, we constructed a partograph for nulliparous based on this data.

Consistent with clinical experience, our study found that cervical dilation typically accelerates as labor progresses. It is normal not to observe significant changes in cervical dilation within the first 3 hours (180 minutes) of early labor. Our study suggested that no obvious inflection point appeared in the labor curve of nulliparous, aligning with a previous study on Chinese nulliparous women [18]. However, this result differs from a prior study on Japanese parturients, which identified a clear acceleration in cervical dilation occurring at 6 cm in nulliparous women [17]. Furthermore, consistent with previous study, our study found that a significant acceleration in cervical dilation should be observed after reaching more than 5 cm in multiparous [17]. In contemporary obstetric practice, this nonlinear relationship should be considered when defining labor arrest in both nulliparous and multiparous.

Our findings suggested that the active phase of cervical dilation usually did not begin until 5 cm or later, even if pregnant women with an active phase were characterized by a steep labor curve in the late first stage. This is similar to Zhang’s results and recent WHO recommendations but differs from the previous view that the active phase begins before 4 cm [1, 13, 15]. These results emphasize that determining labor protraction and arrest in pregnant women should not rely solely on the study-defined average starting point or duration of labor. Recent studies have also questioned the established correlation between cervical dilation and the length of the first stage of labor, as well as the definitions of labor arrest [9, 22]. Given the evolving demographic characteristics of the modern obstetric population, it is crucial to distinguish the typical onset of the active phase from the clinical diagnosis of labor arrest [21]. As long as maternal and infant indicators remain within acceptable ranges and the delivery progresses normally, women should be allowed to continue with the labor process [15].

Furthermore, we conducted a sensitivity analysis using the target population, which included women in main analyses (referred to as the "study population") and women with complicated outcomes (cesarean delivery during the second stage, postpartum hemorrhage, 3rd or 4th degree perineal lacerations, and adverse neonatal outcomes). The aim was to mitigate selection bias during the extraction process and verify the generalizability of labor patterns observed in the main analysis. Consistent with previous study [23], our findings suggested that nulliparous women with complicated outcomes experienced a longer progression of the first stage compared to those without complicated outcomes. Additionally, our study found that nulliparous women in the target population had a longer duration at the 95th centile compared to nulliparous women without complicated outcomes, which is in line with findings from Lundborg et al. [10]. However, the differences in duration at the 95th centile in our study were shorter than those reported in Lundborg’s study (10 minutes vs. 2 hours). This discrepancy may be attributed to Lundborg’s study including all women who underwent cesarean deliveries, whereas our study only included women who had cesarean deliveries during the second stage of labor. Considering labor progression estimates that include women with cesarean delivery and adverse neonatal outcomes may offer more accuracy in the clinical setting than those limited to normal outcomes alone. Further research is warranted to explore labor progression while considering women with complicated outcomes using larger sample sizes in China.

The partograph provides a visual representation of labor, aiming to assess its progression for improved objective management [24]. According to our partograph, midwives can carefully monitor labor progression when the curve is on the left side of the dystocia line. Thorough assessments, considering factors such as oxytocin use, which is linked to preventable adverse perinatal outcomes [25], and primary cesareans, are deemed necessary only if the labor curve crosses the dystocia line. This controlled approach may effectively prevent the overuse of obstetric interventions. Our partograph is poised to assist physicians in better managing labor in Asian women.

### Limitations

Several limitations should be considered in this study. First, due to the high frequency of interventions such as cesarean delivery and instrumental induction of labor in contemporary obstetric practice and the increasing frequency of non-full-term deliveries, only about half of the population has a full-term, spontaneous, vaginal delivery. Second, the data used for this study were collected from only one tertiary hospital in China, and our sample size is small compared to the number of pregnant women in China. Therefore, the study sample may not be representative. Further research is needed to extract data from multiple centers to ensure more representative results are achieved. Third, consistent with a previous similar study [18], a very low percentage (less than 5%) of parturients in our analysis received epidural analgesia, potentially affecting the natural progression of labor. Therefore, the results should be interpreted within the context of contemporary obstetric practice. Although our sample results align with those of Western populations with higher epidural analgesia rates, we recommend further research to compare labor progression differences between pregnant women with high and low rates of epidural analgesia in the Chinese population. Fourth, Due to the lack of precise data about cervical dilation in relation to oxytocin use and amniotomy documented in the medical records, we refrained from comparing the initial cervical dilation in our subgroup analyses.

## Conclusions

The current study found that, in contemporary Chinese nulliparous, the threshold separating the latent and active phases on the labor curve occurs when cervical dilation is 5 cm or higher. It is normal not to observe significant changes in cervical dilation within the first 180 minutes of early labor. We hope that our findings can provide insights for reassessing the labor and delivery process in contemporary obstetric populations, including Chinese obstetric practice.

## Supporting information

S1 Data (XLSX)

S1 File Labor pattern. (ZIP)
