# Supplementary material for: Labor patterns of spontaneous first-stage labor in Chinese women with normal neonatal outcomes
Source: PLoS One. 2024 Jul 3;19(7):e0305243. doi: 10.1371/journal.pone.0305243 (PMC11221650; doi:10.1371/journal.pone.0305243)
Supplement: S1 File — (ZIP) [file pone.0305243.s002.zip › Supplemental Materials/S1 Table.pdf]

S1 Table. Characteristics of the study population by parity and oxytocin augmentation (N = 2,689).

	Nulliparous with oxytocin (n=278)	Multiparous with oxytocin (n=141)	P-value	Nulliparous without oxytocin (n=1200)	Multiparous without oxytocin (n=1070)	P-value
Maternal age (mean ± SD, years)	28.5 ± 3.0	32.9 ± 4.2	<0.001	28.3 ± 3.3	32.4 ± 4.2	<0.001
Maternal weight (mean ± SD, kg)	69.1 ± 8.1	67.8 ± 7.4	0.092	67.8 ± 7.9	67.3 ± 7.0	0.141
Maternal height (mean ± SD, cm)	161.2 ± 4.8	162.3 ± 4.0	0.023	161.0 ± 4.6	160.6 ± 4.4	0.039
BMI at admission (mean ± SD, kg/m^2)	26.6 ± 2.7	25.7 ±2.5	0.002	26.2 ± 2.8	26.1 ± 2.3	0.535
Cervical dilation at admission (cm) [median, 10th, 90th centiles]	3 [2, 4]	3 [1, 4]	0.939	3 [1, 4]	3 [2, 4]	<0.001
Epidural analgesia (%)	6.8	0.7	0.003	3.3	1.1	0.001
Amniotomy (%)	42.8	28.4	0.004	30.3	26.5	0.051
Total number of vaginal exams in 1st stage [median, 10th , 90th centiles]	4 [3, 6]	4 [3, 5]	<0.001	4 [3, 6]	4 [3, 5.5]	<0.001
Gestational age at delivery (mean ± SD, weeks)	39.3 ± 1.0	39.2 ± 1.1	0.363	38.9 ± 1.0	38.8 ±1.0	<0.001
Birthweight (mean ± SD, grams)	3335 ± 356	3360 ± 388	0.520	3260 ± 326	3342 ± 341	<0.001
