# Supplementary material for: Labor patterns of spontaneous first-stage labor in Chinese women with normal neonatal outcomes
Source: PLoS One. 2024 Jul 3;19(7):e0305243. doi: 10.1371/journal.pone.0305243 (PMC11221650; doi:10.1371/journal.pone.0305243)
Supplement: S1 File — (ZIP) [file pone.0305243.s002.zip › Supplemental Materials/S5 Table.pdf]

S5 Table. Characteristics of the target population and parturient with complicated outcomes by parity ($N = 3,185$).

	Nulliparous in target population (n=1645)	Multiparous in target population (n=1292)	P-value	Nulliparous with complicated outcomes (n=167)	Multiparous with complicated outcomes (n=81)	P-value
Maternal age (mean ± SD, years)	28.4 ± 3.3	32.5 ± 4.2	<0.001	28.7 ± 3.7	32.9 ± 3.7	<0.001
Maternal weight (mean ± SD, kg)	67.8 ± 8.0	67.5 ± 7.1	0.224	65.9 ± 7.7	69.1 ± 7.5	0.002
Maternal height (mean ± SD, cm)	160.9 ± 4.7	160.8 ± 4.4	0.781	159.6 ± 4.7	161.4 ± 5.0	0.004
BMI at admission (mean ± SD, kg/m^2)	26.2 ± 2.8	26.1 ±2.3	0.179	25.9 ± 2.8	26.5 ± 2.3	0.099
Cervical dilation at admission (cm) [median, 10th, 90th centiles]	3 [1, 4]	3 [2, 4]	0.035	3 [2, 4]	3 [2, 4]	0.031
Oxytocin use (%)	19.7	11.4	<0.001	27.5	7.4	<0.001
Epidural analgesia (%)	3.9	1.0	<0.001	3.6	0.0	0.181
Amniotomy (%)	32.6	26.6	<0.001	32.9	24.7	0.185
Total number of vaginal exams in 1st stage [median, 10th , 90th centiles]	4 [3, 6]	4 [3, 6]	<0.001	4 [3, 7]	4 [3, 6]	0.094
Gestational age at delivery (mean ± SD, weeks)	39.0 ± 1.0	38.8 ± 1.0	<0.001	38.9 ± 1.0	38.9 ± 1.1	0.620
Birthweight (mean ± SD, grams)	3270 ± 358	3361 ± 375	<0.001	3234 ± 531	3621 ± 616	<0.001
