# Supplementary material for: Labor patterns of spontaneous first-stage labor in Chinese women with normal neonatal outcomes
Source: PLoS One. 2024 Jul 3;19(7):e0305243. doi: 10.1371/journal.pone.0305243 (PMC11221650; doi:10.1371/journal.pone.0305243)
Supplement: S1 File — (ZIP) [file pone.0305243.s002.zip › Supplemental Materials/S3 Table.pdf]

S3 Table. Duration of labor (in minutes) for nulliparous and multiparous with and without oxytocin augmentation.

Cervical dilation (cm)	Nulliparous with oxytocin		Multiparous with oxytocin		Nulliparous without oxytocin		Multiparous without oxytocin	
	n	Median (95th centile)	*n*	Median (95th centile)	*n*	Median (95th centile)	*n*	Median (95th centile)
3-4	232	60 (210)	--	--	913	45 (180)	--	--
4-5	264	30 (155)	134	10 (60)	1112	30 (120)	1014	10 (50)
5-6	272	20 (120)	139	10 (30)	1164	20 (90)	1047	10 (30)
6-7	277	20 (80)	141	5 (20)	1188	15 (60)	1060	5 (20)
7-8	277	15 (60)	141	5 (15)	1191	10 (60)	1062	5 (20)
8-9	277	15 (70)	141	5 (10)	1192	10 (45)	1063	5 (15)
9-10	277	15 (60)	141	5 (20)	1194	10 (40)	1063	5 (15)
3-10	232	220 (550)	--	--	913	168 (490)	--	--
4-10	264	150 (400)	134	45 (170)	1112	110 (350)	1014	45 (120)
2nd stage with epidural	19	68 (191)	1	14 (14)	39	75 (125)	12	22.5 (79)
2nd stage without epidural	259	53 (139)	140	20 (75)	1161	50 (123)	1058	16 (57)
