# Supplementary material for: Labor patterns of spontaneous first-stage labor in Chinese women with normal neonatal outcomes
Source: PLoS One. 2024 Jul 3;19(7):e0305243. doi: 10.1371/journal.pone.0305243 (PMC11221650; doi:10.1371/journal.pone.0305243)
Supplement: S1 File — (ZIP) [file pone.0305243.s002.zip › Supplemental Materials/S4 Table.pdf]

S4 Table. Duration of labor (in minutes) for nulliparous and multiparous with and without amniotomy.

Cervical dilation (cm)	Nulliparous with amniotomy		Multiparous with amniotomy		Nulliparous without amniotomy		Multiparous without amniotomy	
	n	Median (95th centile)	*n*	Median (95th centile)	*n*	Median (95th centile)	*n*	Median (95th centile)
3-4	388	60 (245)	--	--	757	30 (140)	--	--
4-5	456	40 (180)	310	15 (60)	920	20 (120)	838	10 (40)
5-6	475	30 (120)	319	10 (35)	961	15 (60)	867	10 (30)
6-7	482	20 (80)	321	5 (30)	983	10 (60)	880	5 (20)
7-8	482	15 (60)	322	5 (20)	986	10 (50)	881	5 (20)
8-9	482	15 (60)	322	5 (20)	987	10 (45)	882	5 (15)
9-10	482	15 (50)	322	5 (15)	989	10 (40)	882	5 (15)
3-10	388	240 (600)	--	--	757	140 (430)	--	--
4-10	456	163.5 (455)	310	55 (150)	920	95 (325)	838	40 (120)
2nd stage with epidural	35	68 (142)	8	22.5 (79)	23	76 (125)	5	14 (49)
2nd stage without epidural	447	55 (127)	316	17 (59)	973	48 (127)	882	17 (58)
