# Supplementary material for: Labor patterns of spontaneous first-stage labor in Chinese women with normal neonatal outcomes
Source: PLoS One. 2024 Jul 3;19(7):e0305243. doi: 10.1371/journal.pone.0305243 (PMC11221650; doi:10.1371/journal.pone.0305243)
Supplement: S1 File — (ZIP) [file pone.0305243.s002.zip › Supplemental Materials/S2 Table.pdf]

S2 Table. Characteristics of the study population by parity and amniotomy (*N* = 2,689).

	Nulliparous with amniotomy (*n*=482)	Multiparous with amniotomy (*n*=324)	*P*-value	Nulliparous without amniotomy (*n*=996)	Multiparous without amniotomy (*n*=887)	*P*-value
Maternal age (mean ± SD, years)	28.3 ± 3.2	31.9 ± 4.1	<0.001	28.3 ± 3.3	32.6 ± 4.2	<0.001
Maternal weight (mean ± SD, kg)	68.0 ± 8.1	67.7 ± 7.3	0.663	68.1 ± 7.9	67.2 ± 6.9	0.016
Maternal height (mean ± SD, cm)	161.0 ± 4.7	161.3 ± 4.3	0.389	161.0 ± 4.6	160.6 ± 4.4	0.039
BMI at admission (mean ± SD, kg/m^2)	26.2 ± 2.8	26.0 ±2.2	0.262	26.2 ± 2.8	26.1 ± 2.3	0.114
Cervical dilation at admission (cm) [median, 10th, 90th centiles]	3 [2, 4]	3 [2, 4]	0.403	3 [2, 4]	3 [1, 4]	0.004
Oxytocin use (%)	24.7	12.4	<0.001	16.0	11.4	0.004
Epidural analgesia (%)	7.3	2.5	0.002	2.3	0.6	0.003
Total number of vaginal exams in 1st stage [median, 10th , 90th centiles]	5 [3, 7]	4 [3, 6]	<0.001	4 [3, 6]	4 [3, 5]	<0.001
Gestational age at delivery (mean ± SD, weeks)	39.2 ± 0.9	38.9 ± 0.9	<0.001	38.9 ± 1.0	38.8 ± 1.0	0.140
Birthweight (mean ± SD, grams)	3333 ± 325	3399 ± 338	0.005	3246 ± 334	3324 ± 347	<0.001
